# Discovering MXenes, the building blocks for future technology: an interview with Yury Gogotsi

**DOI:** 10.1093/nsr/nwaf431

**Published:** 2025-10-13

**Authors:** Meikang Han

**Affiliations:** Institute of Optoelectronics & College of Future Information Technology, Shanghai Frontiers Science Research Base of Intelligent Optoelectronics and Perception, and State Key Laboratory of Photovoltaic Science and Technology, Fudan University, China

## Abstract

*MXenes, a large and still expanding family of 2D transition metal carbides and nitrides, provide abundant building blocks with unique properties for advancing low-dimensional materials and devices. Since their discovery in 2011, hundreds of MXene compositions have been synthesized and shown tunable electronic, optical, mechanical and electrochemical properties, leading to applications ranging from optics, optoelectronics and wireless communication to energy storage, sensing and biomedicine. In particular, the unusual interactions of MXenes with electromagnetic waves over ultraviolet, visible, infrared, terahertz and microwave ranges offer tremendous opportunities in emerging information technology and electromagnetic protection. Nowadays, after more than a decade of development, MXenes have also reached the critical stage of industrial transformation in addition to further fundamental exploration*.

*NSR spoke to the leading inventor of MXenes—Distinguished University Professor Yury Gogotsi, the Charles T. and Ruth M. Bach Endowed Chair in the Department of Materials Science and Engineering at Drexel University, Director of the A. J. Drexel Nanomaterials Institute, a Fellow of the National Academy of Inventors, the World Academy of Ceramics, the European Academy of Sciences, Academia Europaea and many professional societies, the Citations Laureate in Physics by Clarivate, and recipient of numerous awards*.

## Teaser

The expanding MXene family offers a diverse array of fundamental building blocks, advancing low-dimensional materials and devices across multiple domains, including energy storage, electromagnetic protection, biomedicine, optoelectronics and beyond.

## THE BIRTH AND DEVELOPMENT OF MXENE

***NSR*:** Can you recall the story behind the birth of MXene in 2011, including how the name ‘MXene’ came about?

***Gogotsi*:** It all started from the idea to use Ti_3_SiC_2_ MAX phase as a material for Li-ion battery anodes. My group worked on materials for electrochemical energy storage. I abandoned an attempt to enter the field of silicon anodes by filling templated carbon nanotubes with Si nanoparticles, and was looking for new materials for battery anodes. Ti_3_SiC_2_, which was introduced to the world as a ‘ductile ceramic’ by my Drexel colleague, Professor Michel Barsoum, looked like a promising candidate. It had layers of Si connected by layers of metallically conductive titanium carbide. We worked with it to selectively extract Si and Ti to make layered porous carbide-derived carbon, and I was very familiar with the material. Back-of-the-envelope calculations suggested its attractive capacity, which could exceed that of graphite. Density functional theory (DFT) calculations done by my PhD student Murat Kurtoğlu suggested a reasonably low energy barrier for Li ions to enter the structure. Having those preliminary calculations, I approached Michel Barsoum and suggested submitting a proposal in response to a call from the U.S. Department of Energy BATT program.

Our proposal was funded, and Michael Naguib, a student attracted to Drexel by Michel, was offered a position on the project. He was number one in my graduate thermodynamics class, so I had a lot of confidence in him. He started experiments to get Li into MAX while we were searching for the second student to join the project. However, the start was not successful. We didn’t account for the strain introduced by Li insertion, so Michael failed to introduce any significant amount of Li, and the capacity was too low for any practical applications. It took us several years to show that nanostructuring is required to have MAX phases accessible to Li ions, and still, they have not become competitive with Si anodes. After failing to achieve the necessary capacity

**Figure 1:**
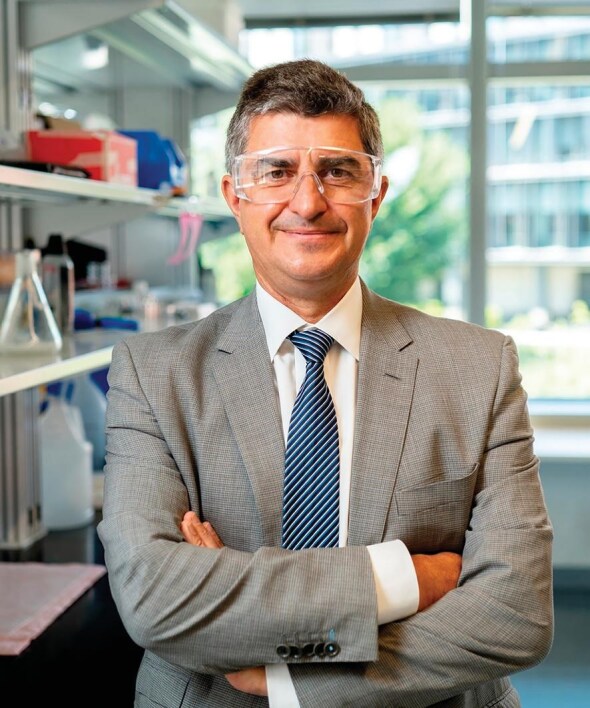
Yury Gogotsi is one of the most cited researchers in the world—ranked 21st globally among all scientists across all disciplines in 2024 by the latest Stanford list based on Scopus data *(courtesy of Professor Yury Gogotsi).*

The history of MXene shows that the path to discovery goes through many failures. However, people who persist are the ones who celebrate success at the end.

—Yury Gogotsi

values, we decided to make space for Si by partial etching of MAX, similar to what we did to produce carbide-derived carbon, but removing just one component. Again, we failed. Si was selectively etched by another of my PhD students, Mohamed Alhabeb, about 6 years later. However, Michael didn’t give up. He tried various etchants and MAX phases until he etched away Al from Ti_3_AlC_2_, producing the first MXene. Since we removed the ‘A’ layer from MAX, the MX layer was left. To show its two-dimensionality, we added the suffix ‘ene’, like in graphene, silicene, borophene and other 2D materials. The rest is history.

You can read the Preface to the first MXene book, *2-D Metal Carbides and Nitrides (MXenes): Structure, Properties and Applications* (there are dozens now) for a more complete story—*The Beginning of MXenes: The Story of the Discovery as Told by the Inventors*. We wrote it together with Michael Naguib and Michel Barsoum so as not to miss any details. The history of MXene shows that the path to discovery goes through many failures. However, people who persist are the ones who celebrate success at the end.

***NSR*:** What are the key performance advantages of MXenes? Did you anticipate from the very beginning that it would become a ‘star material,’ sparking research fervor across so many fields?

***Gogotsi*:** We knew from the beginning that the MXene family was going to be very large. We could project some properties of bulk carbides and MAX phases to MXenes. However, we could not predict some of the properties, such as strong interactions with electromagnetic radiation over a very wide frequency range, anomalously low thermal conductivity combined with metallic electrical conductivity, a wide range of infrared emissivities etc. Those unexpected properties opened the path to many important applications of MXenes. We certainly expect many more discoveries ahead.

***NSR*:** From 2011 to the present, what have been the major research breakthroughs related to MXenes? What are the most exciting current research directions?

***Gogotsi*:** There have been too many to list. Let me go over some of the most important findings. In 2012, the publication of a dozen MXene compositions opened a new class of 2D materials. The next breakthrough was the delamination of multilayer MXenes that allowed the preparation of colloidal solutions of single-layer flakes, the manufacturing of MXene films, and the study of properties of both single flakes and assembled films. In 2013–16, high conductivity and energy storage applications were the focus of MXene research. We demonstrated ultrahigh-rate performance in supercapacitors, which was combined with record values of volumetric capacitance. In 2016, the outstanding electromagnetic interference (EMI) shielding efficiency of Ti_3_C_2_T*_x_* was discovered. Our paper was the first ever publication on this subject in *Science* (or any similarly high-impact journal). It showed that MXenes could greatly outperform graphene and other 2D materials. High shielding effectiveness with flexibility and processability opened opportunities for applications in wearables, stealth technology and flexible electronics. This paper became the most cited paper in the entire EMI field within 5 years of publication and has been cited over 5000 times to date. It also attracted attention to the extreme properties of MXenes, and researchers started exploring MXenes in a large variety of other applications.

Breakthroughs in MXene synthesis by using etching in molten salts, transformation of other 2D materials, and direct vapor phase synthesis have enabled the production of hundreds of new materials now, with the potential for thousands more in the future. Still, so far, only a fraction of the theoretically possible MXenes have been synthesized. Many predicted compositions (beyond Ti-, Nb- and Mo-based) remain unexplored. Tailored surface chemistry beyond O, OH and F could drastically change electronic, catalytic and stability properties. Experimental verification of the predicted topological conductors, semiconducting and magnetic MXenes, which are still mostly theoretical, is needed. Only recently, biocompatibility and toxicity levels of the most common MXenes have been established. The properties of other MXenes are in early stages of exploration. Some of the first real commercial applications are emerging, but the market is still young.

***NSR*:** At what stage is the development of MXenes’ properties currently? How much further potential do you believe remains untapped?

***Gogotsi*:** MXenes were first reported in 2011. Initial excitement focused on their novel 2D structure, conductivity and hydrophilicity, as well as electrochemical properties needed for energy storage applications. The ‘peak of inflated expectations’ has not even been reached yet. Current research is addressing challenges in scalable, environmentally friendly and inexpensive synthesis, control of surface terminations, increasing environmental stability (overcoming oxidation, hydrolysis and degradation in aqueous environments) and integration into devices. MXenes’ properties are well understood for some uses, but limitations remain. There are many gaps in understanding optical and plasmonic properties, interaction of MXenes with ionizing radiation, quantum properties at low temperatures, etc. Moreover, only properties of Ti_3_C_2_T*_x_* have been widely studied. We lack information about the properties of many recently synthesized MXenes. What is important is that MXenes have demonstrated unique combinations of properties (water dispersibility with metallic conductivity, very low thermal conductivity combined with low infrared emissivity, ionic and high electronic conductivity in the same materials, etc.) that make them attractive for many applications.

Creating ionically and electronically conducting (ionotronic) ultra-strong materials, making ultrathin transparent electron transport layers and breaking the Wiedemann–Franz law to make ultrathin thermal insulation led to major progress in advanced technologies.

In summary, MXene property development is in a maturing research stage, but not yet fully industrialized, similar to where graphene was around a decade ago. MXenes’ enormous potential remains largely untapped, especially in new complex compositions, surface terminations, scalable processing and translation into commercial technologies.

MXenes’ enormous potential remains largely untapped, especially in new complex compositions, surface terminations, scalable processing, and translation into commercial technologies.

—Yury Gogotsi

**Figure 2:**
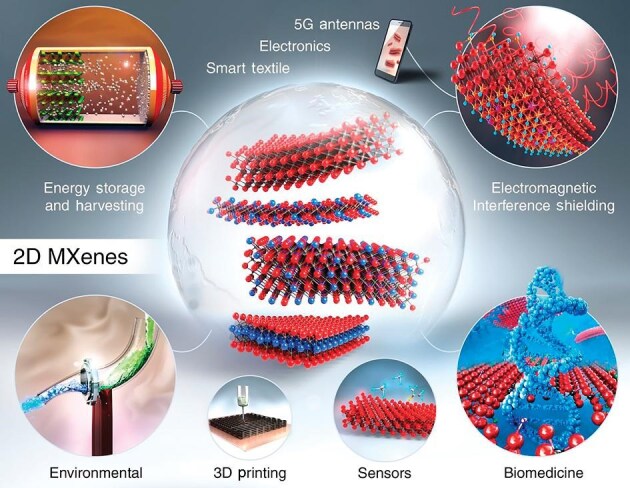
MXenes and their potential applications [reprinted with permission from *Science* (VahidMohammadi A, Rosen J, Gogotsi Y. *Science* 2021; 372: 6547)].

## CHALLENGES IN PRACTICAL APPLICATIONS

***NSR*:** In which field do you think MXenes will achieve large-scale commercial application first? Approximately how long might that take?

***Gogotsi*:** MXenes are already finding applications in EMI shielding. Applications in communication technology, electrophysiology and wearables are moving from proof of concept to performance benchmarking and prototype demonstrations. They are on the way to commercialization. The demand for quality MXenes exceeds the supply at the moment, at least in the U.S.A. Therefore, large-scale applications are currently limited by the production volume. There are companies in the U.S.A, Europe and Asia that make and sell MXenes, but the manufacturing is still limited to batches from hundreds of grams to kilograms. At least a few years will be needed to take it to continuous processes and multi-ton production.

***NSR*:** What are the main challenges in making MXenes practically viable, and what strategies are being employed to address them?

***Gogotsi*:** That’s a key question—MXenes are extremely promising, but several challenges limit their path to widespread practical applications. Large-volume, low-cost and environmentally friendly manufacturing is certainly a challenge. Current production is costly, with batch-to-batch variability in quality, flake size and surface chemistry. However, with numerous new synthesis methods emerging, this is just a matter of time. Current syntheses rely heavily on HF or *in situ* fluoride-based etchants, which are hazardous. Batch processes dominate; continuous, large-scale production is underdeveloped.

Long-term stability of MXenes is another challenge. They can hydrolyze or oxidize in water or humid air, degrading into oxides. We have addressed this challenge by producing stoichiometric MAX phase precursors and MXenes, as well as by optimizing the synthesis protocols. Once processed into films, most MXenes can work for years in the ambient environment.

Better control of surface terminations may be required for some applications. MXenes produced by wet chemical synthesis usually have mixed surface terminations (=O, –OH, –F). This leads to variability in electronic, catalytic and optical properties.

Translating lab-scale processing into square miles of conductive films, robust, reliable and manufacturable devices is the next step. Processing MXenes into composites without losing conductivity or stability at the commercial scale is also needed.

Finally, one needs to establish standard protocols for characterization and storage, as well as conduct life-cycle and techno-economic assessments to prove the competitiveness of MXenes.

***NSR*:** What are the recent focuses of your team regarding both fundamental research on MXenes and their industrial translation?

***Gogotsi*:** My team currently focuses on dry chemical synthesis at elevated temperatures, direct gas phase synthesis of MXenes, and selected applications of interest to our sponsors. On the fundamental side, we are trying to understand the mechanism of MXene formation in the gas phase, direct synthesis of MXenes beyond M_2_CCl_2_, and growth of large-area MXene on substrates parallel to the surface. This will open the path to new electronic applications. We are working to identify applications that can be uniquely enabled by MXenes or their composites, where no other known materials can deliver a comparable performance.

## MATERIALS SCIENCE AND BEYOND

***NSR*:** Besides MXenes, what other emerging materials are you interested in? How do you select your research directions?

***Gogotsi*:** Materials science thrives on novel ideas—whether discovering 2D materials, creating high-entropy MXenes or inventing new applications for our materials. Serendipity is often involved. Ideas often come from listening to conference lectures, reading new papers or discussing with colleagues. One should not be afraid of venturing into new fields. I’m excited about MXenes at the moment, but all my previous shifts from structural ceramics to porous carbons, to semiconductors or nanodiamonds resulted from unexpected discoveries. They were not planned, and serendipity always played a role. Therefore, I cannot say what’s next for me. I keep my eyes open and stay ready to explore new materials as they emerge on my horizon.

***NSR*:** How are new technologies like AI influencing materials science research? Do you have any related attempts or evaluations?

***Gogotsi*:** So far, there has been a lot of hype and very little useful information in terms of materials discovery or optimization. However, AI already helps researchers to be more efficient in writing papers and mining the literature (quickly increasing numbers of papers and patents) to find trends and hidden correlations. It builds large materials databases (Materials Project, etc.) and enhanced by machine learning (ML) models. I do not doubt that much more is to come, so we need to learn how to use AI efficiently now.

AI can predict optimal synthesis parameters (temperature, etching conditions, precursor choice, etc.). Robotic labs and reinforcement learning can enable autonomous synthesis and delamination loops. For example, closed-loop systems may optimize MXene stability. Automated defect detection and classification may be enabled by AI-driven electron microscopy.

The bottlenecks include high-quality, standardized datasets (especially for emerging materials like MXenes), integration of experimental and computational workflows, and interpretability of AI predictions.

Ultimately, AI will reshape materials science research in powerful ways. High-throughput screening of millions of candidate compounds for stability, conductivity, catalytic activity, etc. is not possible manually or by DFT alone. Selection of the structure or composition needed for a desired property (e.g., semiconducting or superconducting MXenes) can be enabled by AI. Deep learning models can predict bandgaps, adsorption energies, optical properties, etc.

***NSR*:** Materials science is a highly active research field. What key qualities do young researchers need to stand out?

***Gogotsi*:** Materials science is an interdisciplinary field, and broad knowledge is needed to succeed. Modern materials challenges—like developing materials for electrochemical energy harvesting and storage, biomaterials or quantum materials—require knowledge across electrochemistry, biology, physics and other domains. Gain a foundation in core materials science areas (e.g., crystallography and thermodynamics) but don’t be afraid to venture into adjacent fields like chemistry or bioengineering; learn computational and AI tools.

Acquire strong experimental and/or computational skills to make, characterize or model materials effectively. One needs an array of simple and advanced techniques, such as electron and optical microscopy, X-ray diffraction, spectroscopy, etc.

Ideas often come from listening to conference lectures, reading new papers, or discussing with colleagues. One should not be afraid of venturing into new fields.

—Yury Gogotsi

Learn the most common ones, but become a true expert in at least one technique. Mastering DFT and molecular dynamics simulations, or data science and ML-based predictions, is useful even if you are not a hard-core computational scientist. Develop your scientific writing skills, learn how to write convincing papers and compelling research proposals, and understand the peer-review process.

Always think about if and why your research matters. There are big materials’ problems related to climate change, energy, biomedicine, future electronics and sustainable development. Aligning your work with pressing real-world issues will make your research more impactful and fundable.

Finally, be patient. The path to discoveries goes through many failures. Don’t hesitate to do risky experiments and explore areas that have not been tackled by others, but keep in mind that developing new materials is often a slow and uncertain process. So, be patient with experiments, flexible with failed hypotheses, and creative with problem-solving.

***NSR*:** How can researchers effectively engage in interdisciplinary collaboration? Do you have any tips or experiences to share?

***Gogotsi*:** Materials research is rarely done in isolation—many teams are diverse and global. Interdisciplinary collaboration is becoming essential, especially in fast-moving fields like MXenes. No single researcher can master all aspects of the MXene enterprise (synthesis, characterization, theory, properties and applications). Here are some practical tips researchers can use to engage effectively:

- First, and most importantly, don’t be afraid to get outside your comfort zone.
- Build networks, look for contacts when presenting at broad national and international conferences, and seek multidisciplinary partnerships.
- Use conceptual models, diagrams or analogies to bridge disciplinary gaps when communicating with researchers outside your field.
- Schedule regular meetings to exchange progress and clarify confusion, which may easily happen.
- Use collaborative tools—share documents in the cloud (Google Docs, Dropbox etc.).
- Be willing to learn outside your field of expertise.
- Avoid territoriality—celebrate joint ownership of discoveries and inventions.
- Encourage students/postdocs to take courses or internships outside their primary field.
- Mixed-background teams (e.g., chemists + materials engineers + computational modelers) often produce the most impactful breakthroughs.

MXene research accelerates from fundamental discovery to applied technology and commercialization. This also means that scientists who do basic research should collaborate with engineers who develop applications and, eventually, companies and investors interested in the commercialization of the new materials and technologies. Without this, there won’t be technological progress.

